# *Vitellogenin *family gene expression does not increase *Drosophila* lifespan or fecundity

**DOI:** 10.12688/f1000research.3975.1

**Published:** 2014-06-10

**Authors:** Yingxue Ren, Kimberly A. Hughes

**Affiliations:** 1 Department of Biological Science, Florida State University, Tallahassee, FL 32306, USA

## Abstract

One of the most striking patterns in comparative biology is the negative correlation between lifespan and fecundity observed in comparisons among species. This pattern is consistent with the idea that organisms need to allocate a fixed energy budget among competing demands of growth, development, reproduction and somatic maintenance. However, exceptions to this pattern have been observed in many social insects, including ants, bees, and termites.  In honey bees ( *Apis* *mellifera*), *Vitellogenin* ( *Vg*), a yolk protein precursor, has been implicated in mediating the long lifespan and high fecundity of queen bees. To determine if *Vg*-like proteins can regulate lifespan in insects generally, we examined the effects of expression of *Apis* *Vg *and *Drosophila CG31150 *(a *Vg*-like gene recently identified as *cv-d*) on *Drosophila* *melanogaster* lifespan and fecundity using the RU486-inducible GeneSwitch system. For all genotypes tested, overexpression of *Vg* and *CG31150 *decreased *Drosophila* lifespan and did not affect total or age-specific fecundity. We also detected an apparent effect of the GeneSwitch system itself, wherein RU486 exposure (or the GAL4 expression it induces) led to a significant increase in longevity and decrease in fecundity in our fly strains. This result is consistent with the pattern reported in a recent meta-analysis of *Drosophila* aging studies, where transgenic constructs of the UAS/GAL4 expression system that should have no effect (e.g. an uninduced GeneSwitch) significantly extended lifespan in some genetic backgrounds. Our results suggest that *Vg-*family genes are not major regulators of *Drosophila* life history traits, and highlight the importance of using appropriate controls in aging studies.

## Introduction

Aging (senescence) is an almost universal process in multicellular organisms, in which organismal function and performance decline with age ^1, 2^. Decreasing fertility and increasing mortality are general hallmarks of aging that are typically accompanied by a declining activity level, altered metabolic rate, and a higher susceptibility to predation, parasites and disease ^3– 5^. Despite its ubiquity, patterns and rates of aging vary enormously among, and within species. A large body of theory and experiment explores the evolutionary causes of this diversity; however, the underlying molecular mechanisms are still poorly understood ^6, 7^.

Evolutionary life history (LH) theory generally assumes that allocation of energy among the competing demands of growth, development, reproduction and somatic maintenance lead to functional trade-offs among these processes ^8, 9^. Consistent with this idea of resource allocation trade-offs, lifespan and fecundity are generally negatively correlated in comparisons among species ^8, 10^, A prominent exception to this pattern occurs in many social insects. In many ants, termites, and bees ^11^, reproductive females are both long-lived and highly fecund relative to other species. For example, queen black garden ants ( *Lasius niger*) can live for at least 28 years, while laying hundreds of eggs per day ^12^, and queen honey bees ( *Apis mellifera*) have a maximum lifespan of 3–5 years while laying thousands of eggs per day ^13^. In contrast, most non-social insects have adult longevity of less than one year, and have lower fecundity than social insect queens ^14^.

Investigations in honey bees suggest that the *Vitellogenin* gene ( *Vg*) produces a yolk protein precursor that is synthesized in the abdominal fat body and acts as an antioxidant and promotes longevity in queen bees ^15^. Differential expression of *Vg* has also been associated with the differences in lifespan between different kinds of worker bees: higher expression is seen in "winter bees" which have a lifespan of 10 months to 1 year, and lower expression in "summer bees" with a lifespan of 30–50 days ^16– 18^. RNAi knockdown of *Vg* expression in workers resulted in lower oxidative stress resistance ^19^ and shorter lifespan ^20^. In contrast, RNAi knockdown of *Vitellogenin*-encoding genes in the nematode *Caenorhabditis elegans* increased survival ^21^ and the expression of genes with similar function in the fruit fly *Drosophila melanogaster* (yolk protein genes) is negatively correlated with lifespan ^22^. The strong support for a role for *Vg* expression in regulating lifespan and fecundity in honey bees, combined with conflicting results from other species, led us to ask if honey bee *Vg* has different functional properties than its homologs in other invertebrates. Specifically, we asked (1) if transgenic expression of honey bee *Vg* in fruit flies can regulate lifespan or fecundity, and (2) if over-expression of a related gene that is endogenous in flies has effects similar that of expression of honey bee *Vg*.

An extensive genetic toolbox allows time and tissue-specific manipulation of gene expression in *D. melanogaster* in ways that are not available in other organisms, and these techniques have been used to characterize the effects of many genes on fly lifespan and fecundity ^23^. For example, manipulations of genes involved in the insulin signaling and the target-of-rapamycin pathways have been causally linked to lifespan regulation in flies (reviewed in ^23, 24^). To our knowledge, however, the effects of *Vg-*family genes on aging-related traits have not been investigated in flies. Unfortunately, *D. melanogaster* lacks a direct homolog of honeybee *Vg*. Instead, gene *CG31150*, recently annotated as *crossveinless d* ( *cv-d*), encodes a *Vg*-like protein that is expressed mainly in the fat body ^25^. The coding sequence for this gene is the most similar among all *Drosophila* genes to *Vg-*encoding genes in honey bee (37% similarity), *C. elegans* (40% similarity), chicken (38% similarity) and zebra fish (38% similar) ^26^. *CG31150* resembles other *Vg* family genes in having an N-terminal *Vitellogenin* N domain, DUF1943 Pfam motifs, and a partial von Willebrand Factor D (VWD) domain near the C terminus ^25^. The biological functions of *CG31150* are largely unknown, but it was recently implicated in lipid transport and bone morphogenic protein (BMP) signaling.

We used the bipartite GAL4/UAS system to manipulate the expression of both honey bee *Vg* and the endogenous *CG31150* gene in flies, and to assess the effects of these manipulations on fly lifespan and fecundity. In this system, expression of a transgene is under the control of a promoter region derived from yeast, the Upstream Activation Sequence (UAS). This promoter region activates transgene expression only when it is bound to the GAL4 protein. Tissue-specific promoters enable spatial control of GAL4 protein production ^27^. Temporal control of transgene expression can be achieved in several ways ^28^, but one of the most convenient methods is using constructs in which the GAL4 DNA binding domain is fused to a progesterone receptor transcriptional activation domain, and is therefore only activated by systemic application of a progesterone-receptor ligand ^29– 31^. We used RU486 (mifepristone)-induced GAL4 drivers (also called the GeneSwitch system) to manipulate our target genes in adult fat body ^32^. A major advantage of this system is that control flies (not exposed to RU486) have exactly the same genotype as transgene-expressing flies, so genetic background differences cannot contribute to differences in phenotype ^33^. We manipulated the expression of both the honey bee *Vg* and *D. melanogaster CG31150* in order to determine whether either or both can regulate lifespan or fecundity in flies. We included two constructs of each gene to account for potential position effects. We also included a series of controls to take into account possible phenotypic effects of RU486 or of the expression of the GAL4 protein ^34, 35^.

## Materials and methods

### *Drosophila* stocks and maintenance

The GeneSwitch driver strain S106 ( *w ^1118^*; P{Switch1}106) and UAS-GFP strain (P{UAS-GFP.VALIUM10}attP2) were obtained from Bloomington *Drosophila* Stock Center. To control for position effects, we used two different transgenic strains for both *Vg* and *CG31150*, with the transgene inserted onto different chromosomes: *CG31150*-2 ( *w ^1118^*, UAS- *CG31150*-2/FM6), *CG31150*-4 ( *w ^1118^*, UAS- *CG31150*-4/TM3), *Vg*-1 ( *w ^1118^*, UAS- *Apis VG*D13-1/FM6), *Vg*-2 ( *w ^1118^*, UAS- *Apis VG*D13-2/TM3). These constructs were created by Eric Spana at the Duke University Model Systems Genomics Core Facility as follows. For the *Vg* constructs, four cDNAs were identified as *Apis Vg* from database searches, and obtained from RIKEN. All four were sequenced on both strands, and one, BH10008D13 was chosen for subsequent cloning as it matched the published sequence best. Site directed mutagenesis was used to remove an ATG from the parental cloning vector (CATTATACGAAGTTA GGGATCAGGCCAAATCGGCCG where the underlined G marks the nucleotide that was changed from a T) so that a NotI/KpnI double digest would excise the *Vg* cDNA from the vector and not contain an incorrect start codon. The NotI/KpnI fragment was then ligated into a NotI/KpnI linearized pUAST. The subsequent clone was verified by sequencing on both strands, and pUAST- *Apis*_ *Vg* was then transformed into *Drosophila* as described below. For the *CG31150* constructs, cDNA GH05619 was available in the “Gold Clone” collection and was obtained from Robin Wharton (Duke University Medical Center). The cDNA was excised from GH05619 using EcoRV & XhoI and ligated into pUAST that had been digested by EcoRI, filled in with Klenow to make a blunt end, and then digested with XhoI to make compatible ends with the insert. The pUAST- *CG31150* plasmid insert was sequenced on both strands. pUAST- *Apis Vg* and pUAST- *CG31150* plasmid inserts were transformed into *w ^1118^* by Model System Genomics of Duke University by standard techniques. All transgenic flies were mapped and balanced. Transgene sequences are shown in Supplementary Table S1.

Overexpression of the target gene in each of the four genotypes was achieved by crossing each transgenic stock with the GeneSwitch strain S106. Strain S106 drives expression specifically in the fat body and also in the digestive system ^32^. This driver has been widely used in aging studies; for example, over-expression of *dfoxo* and *dilp6* using this driver have been shown to increase lifespan ^36, 37^. To produce female flies that expressed the target transgene, virgin females from the S106 stock were crossed to males from each of the UAS-transgenic strains. Half of the female offspring from this cross were reared with RU486-supplemented media throughout adult life (see below), thus inducing transgene expression, but only during the adult stage. The remaining females from each cross were reared on media supplemented only with RU486 vehicle (ethanol), and should not have expressed the transgene. Therefore each group of flies that expressed one of the target transgenes had a genotype-matched control that differed only with respect to whether or not it was fed RU486 or vehicle.

To determine if RU486 exposure (or the GAL4 expression it induced) caused any change in lifespan or fecundity in these flies, we used an additional set of controls. We crossed the S106 driver strain to UAS-GFP flies, and exposed half of the female offspring to RU486 and half only to the ethanol vehicle. GFP is widely used as a reporter in *D. melanogaster*, and is believed to be non-toxic and not to influence endogenous gene expression at any stage during fly development ^38^. Flies were kept on a 12:12 light: dark cycle at 25°C for their entire lifespan.

### Media preparation

An RU486 (Mifepristone, Sigma, St. Louis, MO, USA) stock solution of 25mg/ml was made in 100% ethanol. Appropriate volumes of the stock were diluted with water to reach a final concentration of 65µg/ml, and 300µl of the diluted solution was added onto the surface of standard fly food (1.6% yeast, 0.92% soy flour, 6.76% cornmeal, 4.28% malt, 0.61% agar, 0.25% tegosept, 7.12% corn syrup, 0.61% propionic acid and 77.85% water).

Similar drug concentration and delivery methods have been widely used in aging studies ^32, 35, 36^, and our pilot data indicated that this concentration produced robust expression levels in the range that we desired (5–15 fold increase over non-induced controls). For the control food, an equal amount of 100% ethanol was diluted with water and added to the surface of standard fly food. Each type of food was made fresh twice per week. The vials were allowed to air dry for 48 hours prior to each transfer.

### Lifespan assay

For each genotype tested, female offspring were collected within 24 hours of emergence and split equally into experimental and control groups. For each genotype, 96 females were reared on media supplemented with RU486, and 96 were reared on control media. Flies were housed in standard rearing vials (VWR) at a density of 6 females per vial. All rearing vials were placed randomly in 10 × 10 vial racks to control for possible position effects. The flies were transferred to new media twice per week, and were counted nearly every day. In addition, 3 replicates of 10 females each from each treatment group per genotype were collected separately to be assayed for target gene expression. For these replicates, flies were sampled at 7 days post eclosion; GFP/S106 flies were examined for GFP expression using Zeiss LSM 5 PASCA fluorescent microscopy (Zeiss, Germany), and *Vg*/S106 and *CG31150*/S106 flies were flash frozen on dry ice for quantitative PCR assays.

### Fecundity assay

Females described above were placed in vials with males from a wild-type laboratory strain (described Remolina *et al.* ^39^) when the females were 1 day old. 6 virgin females and 4 males were placed into each vial. The males were removed after 3 days, and females were transferred to new vials. Flies were transferred twice per week. After each transfer, the old vials were kept at 25°C, and all offspring eclosing within 14 days after the adults were removed and counted.

### Quantitative PCR

For each genotype, we pooled 10 flies from each replicate and homogenized whole flies using cordless motors (VWR) and RNase-free pellet pestles (Kimbel Chase). Total RNA was extracted from whole bodies using a PicoPure RNA isolation kit (Arcturus), according to the manufacturer's recommended protocol. RNA purity and quantity were measured using a Nanodrop spectrophotometer (Thermo Scientific). RNA was DNase-treated with a DNA-free RNA kit (Zymo Research) and reverse transcribed using the Superscript III (Invitrogen). We conducted qRT-PCR using SYBR green master mix (Applied Biosystems), and ribosomal gene *Rp49* as an endogenous control. The primer sequences were:

*Rp49*: F ‘TCGAACCAGGCGGGCATATTGT’, R ‘TCGAACCAGGCGGGCATATTGT’;

*Vg*: F ‘AGCTGGTCGGGGCTACGTCC’, R ‘TAAGGGCGTCGGAGGGGACC’;

*CG31150*: F ‘ACGGACACCGACTTCTGTCCCA’, R ‘TCGAACCAGGCGGGCATATTGT’

For transgenic GFP flies (UAS-GFP/S106), expression of GFP was assessed by fluorescent microscopy.

### Statistical analysis

For each transgenic line, we compared Kaplan–Meier (product-limit) survival estimates between RU486- and vehicle-fed flies by using log-rank and generalized Wilcoxon chi-square tests of the homogeneity of survival functions between groups. These tests were conducted using JMP Pro 10 statistical software (SAS Institute Inc., Cary, North Carolina, United States). Log-rank tests are more sensitive to survival differences that occur late in life while Wilcoxon tests are more sensitive to differences earlier in life ^40^. Results of both tests were consistent in all the analyses reported here, so we report only the log-rank test results. To determine if activation of transgene expression by RU486 had different effects in different genotypes, we use used Cox proportional hazard models with predictor variables that included genotype, RU486 treatment, and genotype-by-treatment interaction. A significant interaction in this model would indicate that genotypes responded differently to RU486 treatment. These tests were conducted using the *phreg* procedure of SAS v. 9.3. Flies that died accidentally or escaped during transfers were recorded as censored on the day they died or escaped. For fecundity assays, the mean age-specific fecundity for each vial was calculated by dividing the number of offspring by the number of female parents alive in the vial. We then compared fecundity among genotypes and treatment groups using repeated-measures ANOVA of mean fecundity, with age of the females as the repeated measure. These analyses were conducted in JMP Pro 10.

## Results

### Quantitative PCR confirmed that *Vg* and *CG31150* were overexpressed by the GeneSwitch system

Flies carrying S106 GAL4 driver and each of the target genes, *Vg*-1, *Vg*-2, *CG31150*-2, *CG31150*-4, that were fed on RU486 had a >5 fold increase in target mRNA expression compared with their genetically matched controls ( Table 1). In addition, all 30 GFP/S106 flies that were fed on RU486 showed fluorescent green under the fluorescent microscope while none of their genetically matched controls did (data not shown).

**Table 1.** *Vg* and *CG31150* mRNA expression in each genotype after normalizing to *Rp49*.

Genotype	RU-		RU+		Fold change ^2^
	RQ ^1^	Standard Deviation	RQ ^1^	Standard Deviation	
*Vg*-1/S106	0.71	0.340	11.77	4.333	11.06
*Vg*-2/S106	0.63	0.198	26.68	23.134	26.05
*CG31150*-2/ S106	1.02	0.517	6.48	2.340	5.46
*CG31150*-4/ S106	1.18	0.240	9.97	8.038	8.79

^1^Target gene mRNA expression level is derived by relative quantification (RQ) after normalizing to *Rp49*.

^2^Fold change in target gene expression between RU486-exposed flies and the genetically matched non-fed control.

### Increasing *Vg* and *CG31150* expression does not extend lifespan in *Drosophila*

**Figure 1.**
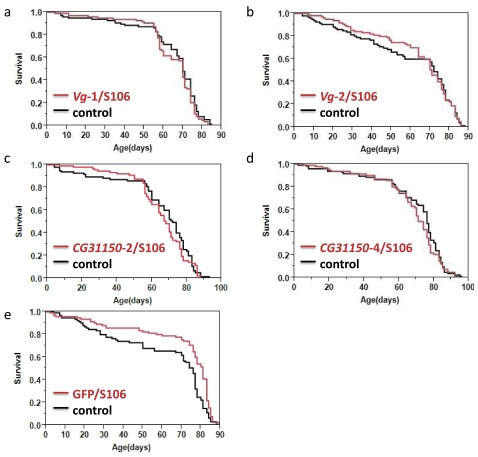
Survival curves for flies either fed RU486 (red) or vehicle only (black, control). No significant lifespan difference was observed between *Vg* or *CG31150* overexpressed flies and their genetically matched control ( **a**– **d**). The negative control GFP/S106 had significantly longer lifespan when fed with RU486 ( **e**).

To test whether *Vg or CG31150* overexpression affected lifespan in *Drosophila*, we examined survival of flies with overexpression of *Apis Vg* and *Drosophila CG31150*. Contradictory to the hypothesis that an increase in the target gene expression would increase lifespan, we observed no significant differences between control and overexpression flies for any of the four experimental genotypes tested ( Table 2, Figure 1a–1d). A proportional-hazards model indicated that there were no significant differences among the four *Vg* and *CG31150* genotypes in their response to transgene activation (χ ^2^ = 0.97, df=3, p=0.81 for genotype-by-RU486 treatment interaction). As an additional control, we assessed whether the RU486 treatment itself was associated with changes in lifespan by comparing RU486-fed and vehicle-fed UAS-GFP/S106 flies. Surprisingly, the RU486-fed flies had a significantly longer lifespan than the controls in this comparison, indicating that RU486 itself (or the GAL4 expression induced by it) had a positive effect on lifespan in this assay ( Table 2, Figure 1e). Thus, the lack of lifespan-extending effects of *Vg* and *CG31150* transgene expression were not due to confounding effects of the method of induction because this method appeared to increase, rather than decrease lifespan in this experiment.

**Table 2.** Median lifespan of target gene over-expressing flies and their genetically matched controls, along with results of log-rank tests of homogeneity of survival curves.

Genotype	n	Median Lifespan (days)	χ ^2^	P
*Vg*-1/S106	89	70	1.903	0.168
Control	87	70		
*Vg*-2/S106	85	71	0.004	0.952
Control	83	72		
*CG31150*-2/S106	78	68	0.777	0.378
Control	77	72		
*CG31150*-4/S106	85	71	1.269	0.260
Control	88	76		
GFP/S106	81	81	11.751	<0.001*
Control	80	76		

To determine if transgene expression had any effect on lifespan in the *Vg* and *CG31150* lines, after accounting for the lifespan-extending effects of RU486 exposure, we compared the effects of RU486 across all genotypes. Proportional hazards models indicated that there was significant heterogeneity among genotypes in their response to RU486 treatment overall (χ ^2^ = 11.2, df=4, p=0.02), with RU486 exposure associated with significantly higher mortality in the *Vg* and *CG3115* transgenic flies than in the GFP transgenic flies in each pairwise comparison (χ ^2^ = 6.5, df=1, p=0.01, hazard ratio = 1.77 for *CG31150-2* vs GFP; χ ^2^ = 7.8, df=1, p=0.005, hazard ratio = 1.85 for *CG31150-4* vs GFP; χ ^2^ = 8.23, df=1, p<0.005, hazard ratio = 1.87 for *Vg-1* vs GFP; χ ^2^ = 4.0, df=1, p<0.05, hazard ratio = 1.56 for *Vg-2* vs GFP). These results suggest that *Vg* and *CG31550* transgene over-expression decrease fly lifespan because (1) GFP over-expression is unlikely to increase lifespan, and in one study was shown to decrease lifespan ^41^, and (2) if transgene expression had no effect in *Vg* and *CG31550* lines, we should have observed a similar increase in lifespan in the RU486-fed flies driven solely by lifespan-extending effects of RU486 and/or GAL4 expression.

### Increasing *Vg* and *CG31150* expression does not increase fecundity in *Drosophila*

**Figure 2.**
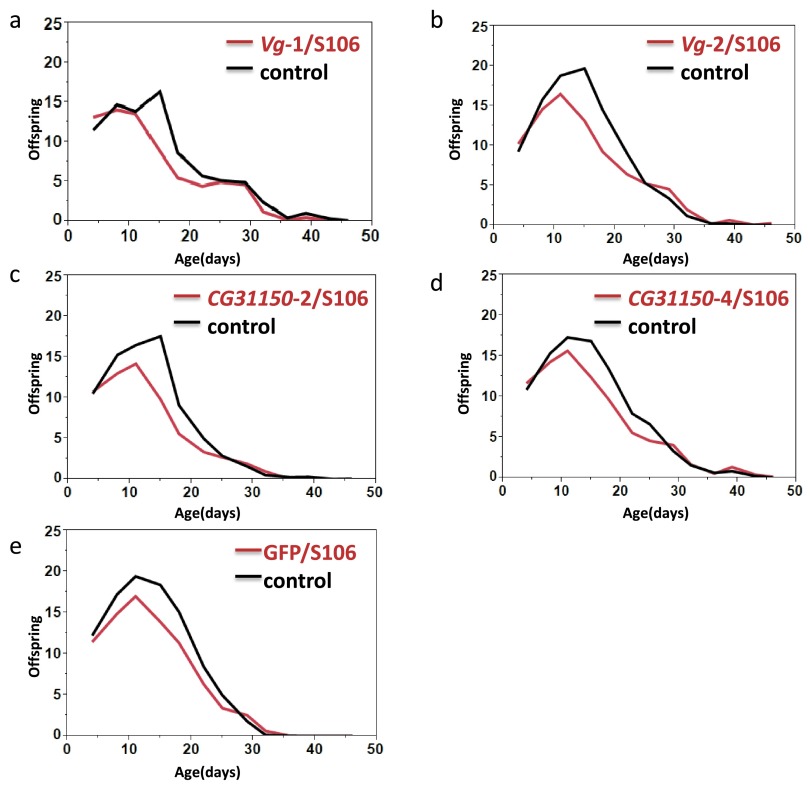
Fecundity experiments on flies either fed on +RU486 (blue) or –RU486 (red). For all of the 5 genotypes tested, the overexpression flies had lower lifetime fecundity than the control. Age × treatment effects were also significant for all phenotypes.

To investigate whether overexpression of *Vg* or *CG31150* increased fecundity in *Drosophila*, we compared age-specific and lifetime fecundity between flies overexpressing these genes and genetically matched controls. We observed a significant overall reduction in fecundity of the RU486-fed flies in all UAS/S106 genotypes, including the control genotype GFP/S106 ( Table 3, Figure 2). The difference between treatments was greatest during mid-life at ages of peak egg-laying, and less at early and late ages (significant age-by-treatment effects were observed in every genotype, Table 3, Figure 2).

**Table 3.** Mean lifetime fecundity targetgene over-expressing flies and their genetically matched controls, along with results of ANOVA tests of treatment and age-by-treatment interaction effects.

Genotype	Lifetime fecundity	Treatment effect		Age × treatment effect	
		F (df)	P value	F (df)	P value
*Vg*-1/S106	70.854	0.353 (1,29)	0.003	5.436 (12,18)	<0.001
Control	84.830				
*Vg*-2/S106	83.101	0.218 (1,30)	0.016	2.646 (12,19)	0.003
Control	97.515				
*CG31150*-2/S106	62.166	0.318 (1,30)	0.004	2.512 (12,19)	0.004
Control	79.311				
*CG31150*-4/S106	81.688	0.195 (1,30)	0.022	2.974 (12,19)	0.001
Control	94.750				
GFP/S106	81.320	0.477 (1,30)	<0.001	2.593 (12,19)	0.003
Control	97.651				

In two-way ANOVA models that included all genotypes, there was no significant genotype-by-treatment interaction (F=0.111, df=4,149, p=0.978) indicating that all transgenic lines, including GFP/S106, responded similarly to RU486 exposure. It therefore appears likely that the decline in fecundity in transgene-expressing flies is caused by RU486 itself or by the expression of GAL4, rather than target-gene overexpression.

*Drosophila* lifespan and fecundity data set **Lifespan raw data:** For each genotype, the death date for each individual was recorded. When a fly died of unnatural causes, the date was recorded under the columns titled “censored”. “Emerged” refers to the date that the individual enclosed. “Cell #” indicates the position of the vial in the 10 × 10 vial racks. **Fecundity raw data:** For each genotype, the number of offspring that enclosed before the day of transfer was recorded, along with the number of mothers that were alive at the time. “Cell #” indicates the position of the vial in the 10 × 10 vial racks. **qPCR raw data:** Target gene mRNA expression level is derived by relative quantification (RQ) after normalizing to *Rp49*. CT, CT mean and CT standard deviation were calculated.

## Discussion

In this experiment, *Vg* and *CG31150* overexpressing flies and their genetically matched controls did not differ significantly in lifespan. However, our negative control GFP/S106 showed significant increase in lifespan when fed RU486. The extended lifespan in the GFP/S106 flies is likely due to the supplement of RU486 in the diet or expression of the GAL4 protein, rather than GFP overexpression. If this lifespan-extending effect of RU486 and/or GAL4 activation also occurred in *Vg-* and *CG31150*-overexpresing lines we investigated, then over-expression of these genes likely had a corresponding negative effect on lifespan.

*Vg* and *CG31150* overexpressing flies showed reduced fecundity comparing with their genetically matched controls. GFP/S106 flies fed with RU486 showed a quantitatively similar reduction in fecundity. These results suggest that over-expression of *Vg* and *CG31150* do not affect fecundity in *Drosophila*. Instead the effect is attributable to RU486, the expression of GAL4, or both.

Tarone *et al.* (2012) ^42^ reported that expression levels of *Drosophila yp* family genes were negatively correlated with longevity among genotypes derived from a natural *D. melanogaster* population. These genes, while unrelated to *Vitellogenin*s, are thought to carry out some functions normally associated with *Vitellogenin*s (e.g., they are believed to comprise the major storage protein in fly embryos) ^26, 43^. Similarly, our results suggest that *Vg*-family gene expression affects fly lifespan. This result contrasts with effects of *Vg* expression in hymenoptera ^15, 16, 19, 44, 45^. The specific pathway through which these genes affect fly lifespan is not known, but our results suggest that it was unlikely due to trade-offs with fecundity, since no increase in fecundity was detected in transgene-overexpressing flies.

In addition to *CG31150*, three other *Vg*-family genes have been described in *D. melanogaster* ^26^, but their effects on aging have not been characterized. These genes include the major hemolymph lipid carrier ( *apolpp*) and regulators of interorgan lipid transport ( *mtp* and *apoLTP*). Products of these genes might therefore play major roles in regulating energy allocation and life history patterns, and future studies of their effects on aging-related traits are warranted.

In addition to testing effects of *Vg* and *CG31150*, our results highlighted the importance of using multiple controls in aging studies. If we had not used the GFP/S106 controls, we would not have identified the effects of RU486 exposure in increasing female fly lifespan and decreasing fecundity, and we would have concluded that expression of our target genes had no effect on lifespan and that they decreased fecundity. Aging-related phenotypes are sensitive to many environmental variables, genetic background, and interaction between genotype and environment. A recent meta-analysis of *Drosophila* aging experiments reported that transgenic constructs of the GAL4/UAS expression system that should have had no phenotypic effects (GAL4 alone, UAS alone, or noninduced GeneSwitch constructs) significantly extended lifespan in the *w ^1118^* genetic background ^46^, consistent with our results. Other studies have reported significant reductions in lifespan caused by RU486 exposure in some genotypes ^35^, suggesting that strain-specific effects of RU486 exposure are common. Experiments using inducible transgenic constructs therefore require multiple sets of controls to reliably assay genetic regulation of aging phenotypes.

## Data availability

F1000Research: Dataset 1. *Drosophila* lifespan and fecundity data set, 10.5256/f1000research.3975.d28311 ^47^

## References

[1] Finch C: Longevity, Senescence, and the Genome. Chicago: University of Chicago.1990 Reference Source

[2] Rose MR: Evolutionary biology of aging. New York: Oxford UP,1991 Reference Source

[3] Tamura T Chiang A Ito N: “Aging specifically impairs amnesiac-dependent memory in *Drosophila*”. *Neuron.* 2003;40(5):1003–11 10.1016/S0896-6273(03)00732-3 14659098

[4] Zerofsky M Harel E Silverman N: “Aging of the innate immune response in *Drosophila melanogaster*”. *Aging Cell.* 2005;4(2):103–08 10.1111/j.1474-9728.2005.00147.x 15771614

[5] Fang M Roscoe F Sigal LJ: “Age-dependent susceptibility to a viral disease due to decreased natural killer cell numbers and trafficking”. *J Exp Med.* 2010;207(11):2369–381 10.1084/jem.20100282 20876312 PMC2964566

[6] Hughes KA: “Mutation and the evolution of ageing: from biometrics to system genetics”. *Philos Trans R Soc Lond B Biol Sci.* 2010;365(1544):1273–79 10.1098/rstb.2009.0265 20308103 PMC2871812

[7] Gems D Partridge L: “Genetics of longevity in model organisms: debates and paradigm shifts”. *Annu Rev Physiol.* 2013;75(1):621–44 10.1146/annurev-physiol-030212-183712 23190075

[8] Stearns SC: The Evolution of Life Histories. Oxford: Oxford UP,1992 Reference Source

[9] Zera AJ Harshman LG: “The Physiology of Life History Trade-offs in Animals”. *Annu Rev Ecol Syst.* 2001;32(1):95–126 10.1146/annurev.ecolsys.32.081501.114006

[10] Roff DA: The Evolution of Life Histories: Theory and Analysis. New York: Chapman & Hall,1992 Reference Source

[11] Keller L Jemielity S: “Social insects as a model to study the molecular basis of ageing”. *Exp Gerontol.* 2006;41(6):553–56 10.1016/j.exger.2006.04.002 16713694

[12] Keller L Passera L: “Fecundity of Ant Queens in Relation to Their Age and the Mode of Colony Founding”. *Insectes Sociaux.* 1990;37(2):116–30 10.1007/BF02224025

[13] Page RE Jr Peng YS: “Aging and development in social insects with emphasis on the honey bee, *Apis mellifera L*”. *Exp Gerontol.* 2001;36(4–6):695–711 10.1016/S0531-5565(00)00236-9 11295509

[14] Keller L Genoud M: “Extraordinary Lifespans in Ants: a Test of Evolutionary Theories of Ageing”. *Nature.* 1997;389:958–960 10.1038/40130

[15] Corona M Velarde RA Remolina S: “ *Vitellogenin*, juvenile hormone, insulin signaling, and queen honey bee longevity”. *Proc Natl Acad Sci U S A.* 2007;104(17):7128–33 10.1073/pnas.0701909104 17438290 PMC1852330

[16] Amdam GV Hartfelder K Norberg K: “Altered physiology in worker honey bees (Hymenoptera: Apidae) infected with the mite Varroa destructor (Acari: Varroidae); a factor in colony loss during overwintering?” *J Econ Entomol.* 2004;97(3):741–747 10.1603/0022-0493(2004)097[0741:APIWHB]2.0.CO;2 15279246

[17] Munch D Amdam GV: “The curious case of aging plasticity in honey bees”. *FEBS Lett.* 2010;584(12):2496–2503 10.1016/j.febslet.2010.04.007 20385132

[18] Munch D Kreibich CD Amdam GV: “Aging and Its modulation in a long-lived worker caste of the honey bee”. *J Exp Biol.* 2013;216(Pt 9):1638–649 10.1242/jeb.078915 23596282 PMC3631978

[19] Seehuus SC Krekling T Amdam GV: “Cellular senescence in honey bee brain is largely independent of chronological age”. *Exp Gerontol.* 2006;41(11):1117–125 10.1016/j.exger.2006.08.004 17052880 PMC2408864

[20] Nelson CM Ihle KE Fondrk MK: “The gene *vitellogenin* has multiple coordinating effects on social organization”. *PLoS Biol.* 2007;5(3):e62 10.1371/journal.pbio.0050062 17341131 PMC1808115

[21] Murphy CT Mccarroll SA Bargmann CI: “Genes that act downstream of DAF-16 to influence the lifespan of *Caenorhabditis elegans*”. *Nature.* 2003;424(6946):277–83 10.1038/nature01789 12845331

[22] Tarone AM Mcintyre LM Harshman LG: “Genetic variation in the Yolk protein expression network of *Drosophila melanogaster*: sex-biased negative correlations with longevity”. *Heredity (Edinb).* 2012;109(4):226–34 10.1038/hdy.2012.34 22760232 PMC3464022

[23] Paaby AB Schmidt PS: “Dissecting the genetics of longevity in *Drosophila melanogaster*”. *Fly (Austin).* 2009;3(1):29–38 10.4161/fly.3.1.7771 19182541

[24] Partridge L Alic N Bjedov I: “Ageing in *Drosophila*: the role of the insulin/Igf and TOR signaling network”. *Exp Gerontol.* 2011;46(5):376–81 10.1016/j.exger.2010.09.003 20849947 PMC3087113

[25] Chen J Honeyager SM Schleede J: “Crossveinless d is a *vitellogenin*-like lipoprotein that binds BMPs and HSPGs, and is required for normal BMP signaling in the *Drosophila* wing”. *Development.* 2012;139(12):2170–176 10.1242/dev.073817 22573617 PMC3357910

[26] Palm W Sampaio JL Brankatschk M: “Lipoproteins in *Drosophila melanogaster*--assembly, function, and influence on tissue lipid composition”. *PLoS Genet.* 2012;8(7):e1002828 10.1371/journal.pgen.1002828 22844248 PMC3406001

[27] Brand AH Perrimon N: “Targeted gene expression as a means of altering cell fates and generating dominant phenotypes”. *Development.* 1993;118(2):401–15 8223268 10.1242/dev.118.2.401

[28] Duffy JB: “GAL4 system in *Drosophila*: a fly geneticist's swiss army knife”. *Genesis.* 2002;34(1–2):1–15 10.1002/gene.10150 12324939

[29] Osterwalder T Yoon KS White BH: “A conditional tissue-specific transgene expression system using inducible GAL4”. *Proc Natl Acad Sci U S A.* 2001;98(22):12596–2601 10.1073/pnas.221303298 11675495 PMC60099

[30] Roman G Endo K Zong L: “P[Switch], a system for spatial and temporal control of gene expression in *Drosophila melanogaster*”. *Proc Natl Acad Sci U S A.* 2001;98(22):12602–607 10.1073/pnas.221303998 11675496 PMC60100

[31] Nicholson L Singh GK Osterwalder T: “Spatial and temporal control of gene expression in *Drosophila* using the inducible GeneSwitch GAL4 system. I. Screen for larval nervous system drivers”. *Genetics.* 2008;178(1):215–34 10.1534/genetics.107.081968 18202369 PMC2206072

[32] Poirier L Shane A Zheng J: “Characterization of the *Drosophila* gene-switch system in aging studies: a cautionary tale”. *Aging Cell.* 2008;7(5):758–70 10.1111/j.1474-9726.2008.00421.x 18691185

[33] Partridge L Gems D: “Benchmarks for ageing studies”. *Nature.* 2007;450(7167):165–67 10.1038/450165a 17994065

[34] Ren C Finkel SE Tower J: “Conditional inhibition of autophagy genes in adult *Drosophila* impairs immunity without compromising longevity”. *Exp Gerontol.* 2009;44(3):228–35 10.1016/j.exger.2008.10.002 18955126 PMC2664319

[35] Shen J Curtis C Tavaré S: “A screen of apoptosis and senescence regulatory genes for life span effects when over-expressed in *Drosophila*”. *Aging (Albany NY).* 2009;1(2):191–211 20157509 10.18632/aging.100018 PMC2806004

[36] Giannakou ME Goss M Jünger MA: “Long-lived *Drosophila* with overexpressed dFOXO in adult fat body”. *Science.* 2004;305(5682):361 10.1126/science.1098219 15192154

[37] Bai H Kang P Tatar M: “ *Drosophila* insulin-like peptide-6 ( *dilp6*) expression from fat body extends lifespan and represses secretion of *Drosophila* insulin-like peptide-2 from the brain”. *Aging Cell.* 2012;11(6):978–85 10.1111/acel.12000 22935001 PMC3500397

[38] Chalfie M: “Green Fluorescent Protein as a Marker for Gene Expression”. *Trends Genet.* 1994;10(5):151 10.1016/0168-9525(94)90088-4 8303295

[39] Remolina SC Chang PL Leips J: “Genomic basis of aging and life-history evolution in *Drosophila melanogaster*”. *Evolution.* 2012;66(11):3390–403 10.1111/j.1558-5646.2012.01710.x 23106705 PMC4539122

[40] Allison PD: Survival Analysis Using SAS System: A Practical Guide. Cary, NC: SAS Institute,1995 Reference Source

[41] Mawhinney RM Staveley BE: “Expression of GFP can influence aging and climbing ability in *Drosophila*”. *Genet Mol Res.* 2011;10(1):494–505 10.4238/vol10-1gmr1023 21476195

[42] Tarone AM McIntyre LM Harshman LG: “Genetic variation in the Yolk protein expression network of *Drosophila melanogaster*: sex-biased negative correlations with longevity”. *Heredity (Edinb).* 2012;109(4):226–34 10.1038/hdy.2012.34 22760232 PMC3464022

[43] Bownes M: “Why is there sequence similarity between insect yolk proteins and vertebrate lipases?” *J Lipid Res.* 1992;33(6):777–90 1512506

[44] Landis GN Abdueva D Skvortsov D: “Similar gene expression patterns characterize aging and oxidative stress in *Drosophila melanogaster*”. *Proc Natl Acad Sci U S A.* 2004;101(20):7663–7668 10.1073/pnas.0307605101 15136717 PMC419663

[45] Brandt BW Zwaan BJ Beekman M: Shuttling between species for pathways of lifespan regulation: a central role for the *Vitellogenin* gene family? *BioEssays.* 2005;27(3):339–346 10.1002/bies.20161 15714554

[46] Ziehm M Piper MD Thornton JM: Analysing variation in *Drosophila* aging across independent experimental studies: a meta-analysis of survival data. *Aging Cell.* 2013;12(5):917–922 10.1111/acel.12123 23795998 PMC3963443

[47] Ren Y Hughes KA: *Drosophila* lifespan and fecundity data set. *F1000Research.* 2014 Data Source 10.12688/f1000research.3975.1 PMC4111121 25110583

