# Supplementary material for: Vitellogenin family gene expression does not increase Drosophila lifespan or fecundity
Source: F1000Res. 2014 Jun 10;3:125. [Version 1] doi: 10.12688/f1000research.3975.1 (PMC4111121; doi:10.12688/f1000research.3975.1)
Supplement: Peer review history

# Peer review: Referee response for version 1

**DOI:** 10.5256/f1000research.4260.r5099

**Published:** 2014-06-24

**Authors:** John Tower

**Affiliations:** 1 Davis School of Gerontology and Department of Biological Sciences, University of Southern California, Los Angeles, CA, USA

Previous reports of a positive correlation between *Vg* gene expression and longevity in the hymenoptera prompted this interesting and timely test of *Vg *gene over-expression in *Drosophila*. The data show that there is no simple strong benefit to life span when over-expressing *Vg* genes in the *Drosophila *adult fat body, using the S1-106 Gene-Switch driver strain. In addition, because a life span increase was observed in a control cross involving over-expression of GFP, the results suggest possible effects of the drug, the activated Gene-Switch transcription factor, the GFP, or the variability of the assay. These results underscore the importance of careful controls for possible life span effects on the conditional gene expression system itself, independent of the specific identity of the gene being over-expressed.

The data are clearly presented and support the conclusions above, however, the generality of the conclusions must be tempered somewhat given the limited scope of the study. 

 

1. In previous *Drosophila *studies life span extension was observed when transgenes were expressed with certain drivers, but not others, including some cases where the drivers tested had similar tissue specificities.  Those results indicate that the precise tissue-specificity and/or level of transgene expression can be important. In the present study the *Vg* transgenes were expressed using a Gene-Switch driver strain that yields expression in only a subset of the fat-body tissues, so it remains possible that over-expression of the *Vg* genes using a driver with more extensive expression pattern in the fat body and/or other tissues might create a life span increase. 
2. The conclusions regarding the possible cause of the life span increase in the control cross are based on the assumption that over-expressed GFP has little or no phenotypic effect.  However the one *Drosophila *study cited reports a significant (negative) effect of GFP expression on aging phenotypes (using different drivers).  If GFP can have negative effects in some tissues it seems possible it might have positive effects when expressed in other tissues (for example, the “negative” effect of killing the IPCs or the germ cells can increase fly life span). For these reasons it is not clear whether the life span increase observed in the present study results from the drug, the activated Gene-Switch, or the GFP. Finally, it should be noted that this result was not replicated. These considerations limit the support for a possible negative life span effect of the *Vg* transgenes as suggested in the Abstract and Discussion.

I have read this submission. I believe that I have an appropriate level of expertise to confirm that it is of an acceptable scientific standard.

# Peer review: Referee response for version 1

**DOI:** 10.5256/f1000research.4260.r5095

**Published:** 2014-06-23

**Authors:** Joel Parker

**Affiliations:** 1 Department of Biological Sciences, State University of New York at Plattsburgh, Plattsburgh, NY, USA

This is a well written, interesting and well executed study showing similarities between honey bee and *Drosophila vitellogenin*, but with neither extending lifespan nor effecting fecundity when over expressed in flies. The study also offers an important lesson in using controls in UAS-GAL4 systems. The purpose was to ask whether the most closely related gene in *Drosophila* to the *vitellogenin* gene in honey bee is also involved in aging. This was a long shot from the level of homology but a question worth asking. The results were basically negative but the authors also found very clear and striking effects in the controls that anyone using the UAS-GAL4 system should be aware of.

There are several points that the authors should consider addressing in their revision:

1. Are all five of the transgenic sets on the same genetic background? It is not clear from the methods where the *w ^1118 ^*flies were sourced and there is no mention of backcrossing. If they came from different sources it is possible that the backgrounds might be different making comparisons across the five less meaningful. Even if the backgrounds are not exactly the same the on/off nature of the UAS-GAL4 system in the same lines still means the effects are real within each pair.
2. Another interpretation of the longevity results is that the honey bee *Vg* and fly homolog both blocked the effect of GAL4 instead of the authors’ interpretation that over expression of both of these genes decrease lifespan. This is an important because it might be overreaching to interpret the results as a shortening of lifespan in general unless one assumes the effects of GAL4 and the *Vg* genes are independent and additive.
3. The authors should start off the discussion with an important positive result they neglected to point out, that both the honey bee and the fly *Vg *genes showed exactly the same effects on both longevity and fecundity. The consistent results from the two *Vg*’s might be saying they share some functions. Hence the previously seen effect on aging is only in honey bees and not flies. I realise that this is a weak supposition given that fecundity was not changed and that only a control effect may have been reversed in the aging experiment, but the fact that both gave exactly the same results is consistent with the central hypothesis that the two have homologous functions.
4. I think there is a mis-wording at the end of the second sentence of the 5 ^th^ paragraph in the Discussion. Remove “ *that they decreased*”. The point is that fecundity was not reduced when you consider the effect seen in the control.
5. ​Early on in the honeybee *vitellogenin* story there was a suggestion that it increased lifespan by acting as an anti-oxidant. If this was the case then one might have expected to see an increase in lifespan in the *Drosophila* as well. The fact that this did not happen suggests that honeybee *vitellogenin* is working in bees in another manner. On the other hand, antioxidants can decrease lifespan in some *Drosophila* genetic backgrounds in which case they see *Vg*’s functioning in exactly the same way in both flies and bees (assuming the GAL4 is additive). Although this is all speculative, it still might be worth going into a bit more detail about what is known and thought about the molecular mechanisms underpinning how *Vg* effects aging in honey bees and more clearly define the *a priori* predictions. 

Summary:

- The title is appropriate
  ​
- Abstract is adequate
- Everything is well explained and presented
- The conclusions are sound and sufficiently conservative, I do make some suggestions above.

I have read this submission. I believe that I have an appropriate level of expertise to confirm that it is of an acceptable scientific standard.
